# Genetic and evolutionary analysis of a new Asia-4 lineage and naturally recombinant canine distemper virus strains from Thailand

**DOI:** 10.1038/s41598-019-39413-w

**Published:** 2019-03-01

**Authors:** Chutchai Piewbang, Araya Radtanakatikanon, Jiratchaya Puenpa, Yong Poovorawan, Somporn Techangamsuwan

**Affiliations:** 1 Department of Pathology, Faculty of Veterinary Science, Chulalongkorn University, Bangkok, 10330 Thailand; 2 Department of Pediatrics, Faculty of Medicine, Chulalongkorn University, Bangkok, 10330 Thailand; 3 Diagnosis and Monitoring of Animal Pathogens Research Unit, Faculty of Veterinary Science, Chulalongkorn University, Bangkok, 10330 Thailand

## Abstract

Canine distemper virus (CDV), a pathogen causing fatal disease in a wide range of carnivores, can be classified into several geographically-related lineages. It is unclear how genetic recombination contributed to the evolution and emergence of the novel CDV strains and the evolutions of these strains are not fully yet investigated. In this study, the complete genome sequences of eight CDV viruses, isolated from domestic dogs in Thailand, were investigated. Interestingly, most of the identified CDV strains (CDV1-3, -5, -8 TH/2014) clustered as a novel Asia-4 lineage, while the CDV4, -6, -7 TH/2014 belonged to the Asia-1 lineage. Recombination analysis revealed that the CDV4 TH/2014 is a putative recombinant virus from the Asia-1 and America-2 parent viruses. In contrast, no recombination events were detected in the Asia-4 lineage, indicating that it is a distinctive lineage. Evolutionary analysis suggested that the CDV Asia-4 lineage had emerged since 1924 and shared common ancestor with the America-2 lineage. Pressure analysis revealed that CDV nucleotides were under negative selection pressure for its rapid adaptation. These findings demonstrate the evolution of CDV Asia-4 lineage and identified the Asia-1 recombination event. The information regarding genetic diversity of CDVs is essential for further CDV’s research and monitoring.

## Introduction

Canine distemper virus (CDV) has been recognized as a contagious pathogen causing canine distemper (CD), a fatal systemic disease afflicting in domestic and wild carnivores. CDV belongs to *Morbillivirus* genus and *Paramyxoviridae* family and possesses a non-segmented, negative sense, single-stranded RNA of 15,690 nucleotides. CDV genome encodes six core-structural proteins; phospho (P)-, nucleocapsid (N)-, large polymerase (L)-, matrix (M)-, fusion (F)- and hemagglutinin (H) proteins^1^. The L, N and P proteins, together with a viral RNA, constitute the ribonucleic protein (RNP). The H and F glycoproteins facilitate viral entry process by binding and fusing to the host cells, respectively^2,3^. The H protein is largely known to have the antigenic variation and the antibodies to H protein are critical for the protective immunity against infection^4^.

Due to its genetic variability, the H gene has been used for determining CDV genetic lineages which are related within the geographic origin that the lineages were detected. Currently, there are at least seventeen major CDV genetic lineages, including the America-1 to -5, Europe Wildlife, Arctic, South Africa, America-1/Europe, South America-1 to -3, Rockborn-like, Asia-1 to -3^5–8^ and a novel CDV lineage, Asia-4, that was recently reported in Thailand^9^, and subsequently in China^10^ and Russia^11^. However, because of its genetic variability, both intra- and inter-genetic recombination among lineages have frequently been reported in highly variable regions^5,12–20^. Thus, these evidences may warrant a caution for interpretation of viral phylogenies based on a single genomic region^21^.

Genetic recombination plays an important role in the evolution of RNA viruses, resulting in the emergence of novel viral lineages. Genetic recombination events are well documented among the paramyxoviruses, particularly in Measles virus (MV), Newcastle disease virus (NDV) and Respiratory syncytial virus (RSV)^17,18,22,23^. For CDV, recent studies on the previously available CDV genomes in GenBank database indicated the recombination events of the CDVs that were discovered since late 1998^5,14,20^. These studies showed that recombination occurred in various regions of CDV genome, particularly in the H gene. Therefore, the classification of CDV strain based on partial or full sequence of the H gene may not reflect the true phylogeny, leading to misinterpreting of its origin. To overcome this burden, this study was aimed to characterize and analyse the whole genome sequence and determine the mechanism(s) of genetic evolution of the Thai CDV viruses, particularly the new Asia-4 and the recombinant Asia-1 CDVs by performing extensive phylogenetic recombination, evolutionary and selective pressure analyses.

## Results

### Genetic, phylogenetic and recombination analyses

**Figure 1:**
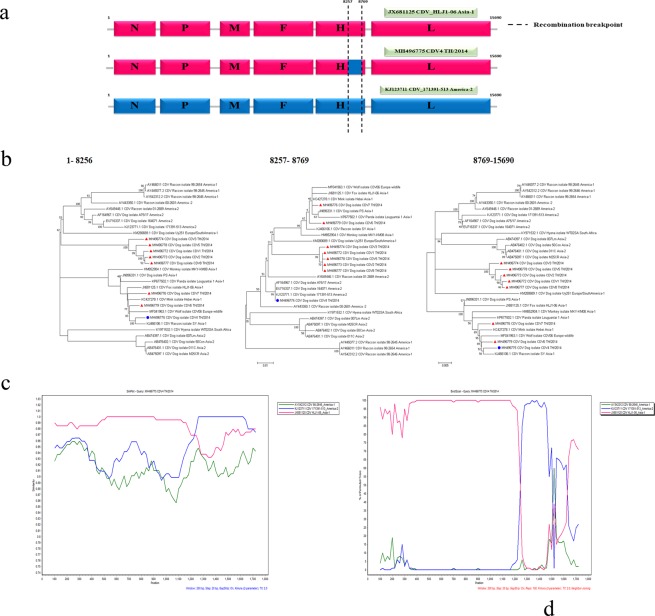
Schematic diagram of genome organization of the potentially recombinant CDV4 TH/2014 strain. A CDV HLJ1-06 Asia-1 strain (accession no: JX688125; pink box) and CDV 171391–513 America-2 strain (accession no: KJ123711; blue box) served as putative major and minor parents. The recombination event is located at nt 8257–8769 in the hemagglutinin gene (**a**). Phylogenetic analysis of CDV’s TH/2014 strains () and the recombinant strain () in the different segments represented by maximum likelihood trees with 1000 bootstraps (**b**). Similarity plot (**c**) and bootscan analysis (**d**) based on complete hemagglutinin gene of the recombinant CDV4 TH/2014 as a query. The y-axis indicates percentage of nucleotide identity and permutated trees for similarity plot and boot scanning, respectively, within a 200 bp-wide window with a 20-bp step size between plots.

All eight Thai CDV isolates, designated as CDV1-8 TH/2014, consist of six core structural genes (Fig. 1a). Pairwise nucleotide identities of all CDVs’ TH/2014 genomes differed from the previously published CDV sequences, ranging from 1.0–7.9% (Supplementary Table S3). Phylogenetic analysis of the whole genome sequences revealed that the CDVs’ TH/2014 segregated into two lineages; one is closely related to the Asia-1 (CDV4, -6, -7 TH/2014) and the other represented another distinctive Asia-4 lineage (CDV1-3, -5, -8 TH/2014) (Fig. 1b). A topology of the phylogenetic tree constructed from most of the genome revealed a high similarity with exception for the H gene fraction (Fig. 1b). The phylogenetic relationships based on nucleotide alignment of full-length genome using neighbor joining and maximum likelihood methods resulted in similar topologies. Recombination analysis of CDVs’ TH/2014 using the RDP program revealed that the CDV4 TH/2014 was a recombinant virus. The recombination breakpoint was identified in the H gene which was supported by statistical methods including RDP, GENECONV, BootScan, Maxchi, Chimaera, SiScan, 3Seq and LARD with *p*-value of 4.715 × 10^−11^, 1.848 × 10^−9^, 4.767 × 10^−11^, 8.613 × 10^−6^, 1.505 × 10^−4^, 1.025 × 10^−8^, 4.072 × 10^−10^ and 3.370 × 10^−10^, respectively. The CDV strain HLJ1-06 (JX681125; Asia-1 lineage)^24^ and strain 171391-513 (KJ123771; America-2 lineage) were identified as major and minor putative parents of the CDV4 TH/2014 (Fig. 1a). Further analysis based on the RDP results were subjected to the similarity plot and bootscan analyses (Fig. 1c,d). The results revealed that the recombinant CDV4 TH/2014 virus had a high nucleotide identity to the CDV HLJ1-06 (JX681125; pink line). However, the nucleotides identity to the CDV 171391–513 (KJ123771; blue line) could be clearly observed at the 3′ end of the H gene. The putative recombination breakpoint of the CDV4 TH/2014 strain was located at nt.8257–8769.

### Evolutionary analysis of canine distemper viruses circulating in Thailand

**Figure 2:**
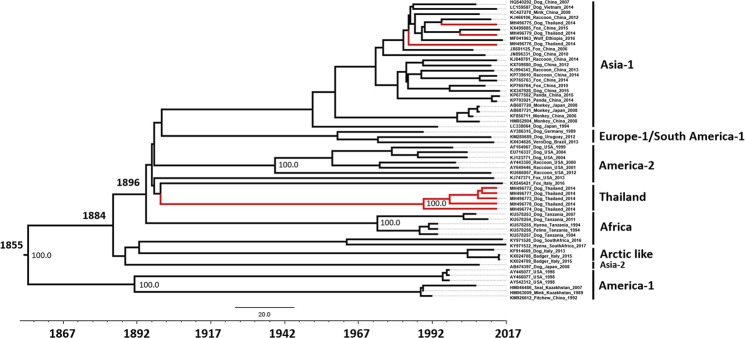
Maximum clade credibility (MCC) tree of 58 full-length CDV genomes sequences. The phylogeny includes 8 CDV genome sequences from Thailand (red line), and 50 full-length CDV sequences available on the GenBank database between 1975 and 2016. The most recent common ancestors (MRCAs) of these strains are indicated. Dates of MRCA existence are shown on the nodes.

**Figure 3:**
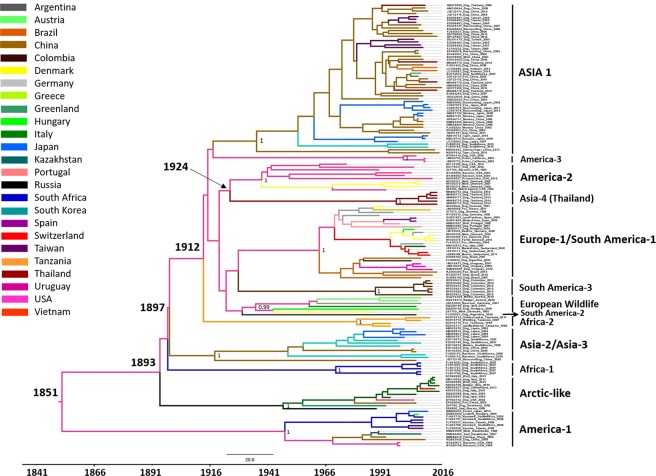
Maximum clade credibility (MCC) tree of 156 full-length hemagglutinin genes of CDV. The phylogeny includes 8 hemagglutinin gene sequences of the Thai CDVs, and 148 full-length H gene sequences available on the GenBank database between 1975 and 2016. The most recent common ancestors (MRCAs) of these strains are indicated. Dates of MRCA existence are shown on the nodes. The tree branches are colored according to geographical locations that the strains were isolated.

Two datasets were utilized to estimate time to the most recent common ancestor (tMRCA), substitution rates and evolutionary history of CDV: a complete genome dataset (58 sequences sampled from 13 countries) and an H gene dataset (156 sequences isolated from 25 countries during 1975–2016, including 8 Thai strains isolated in this study). Maximum clade credibility (MCC) trees for complete genome and H gene datasets showed similar topologies and clustering (Figs 2 and 3). The overall evolutionary rate of the full genome of CDV sequence dataset was estimated at 2.46 × 10^−4^ substitutions/site/year [95% HPD: 2.05–2.90 × 10^−4^]. The tMRCA of CDV cluster existence was estimated to be in 1855 [95% HPD:1827–1879].

As the number of complete CDV genome sequences available in the database was limited, and most of the CDV strains were categorized based on its H gene, we further analysed the CDV evolution based on the available H gene sequence. The CDV H gene sequences were clustered based from the previously reported clades^25^. The phylogenetic tree of CDV H gene revealed the overall evolutionary rate of 3.50 × 10^−4^ substitutions/site/year [95% HPD: 2.83–4.17 × 10^−4^] and the predicted MRCA was estimated in 1851 (95% HPD: 1816–1882) (Fig. 3). The CDVs’ TH/2014 viruses were divided into two clades. The CDV1-3, -5 and -8 TH/2014 were uniquely clustered as a novel divergent clade with node high support values (posterior probability value, PP > 0.9) which was proposed as a new Asia lineage. The date of the MRCA of the new Asia cluster existence was estimated to emerge in 1924 (95% HPD: 1906–1939) which diverged from the North America-2 lineage. Although the CDV4, -6 and -7 TH/2014 viruses belong to the Asia-1 strain, the CDV4 TH/2014 seem to be distinctive from other lineages.

### Selective pressure analysis on CDV evolution

The non-synonymous (dN) and synonymous (dS) ratio were achieved from the alignment of the six individual genes from the available CDV genomes. SLAC, FEL and FUBAR analyses indicated that the overall CDV gene has undergone a negative selective pressure, reflecting dN/dS < 1 (Supplementary Table S4). Nevertheless, the potentially positive selection sites were also evident in all, genes with the exception of M and with higher frequencies in the F and H genes.

### Virus isolation of CDVs’ TH/2014

All eight CDV strains were isolated from CDV infected dogs. The viruses induced prominent cytopathic effect (CPE) with stellate cell formation in Vero-DST cell line (Supplementary Fig. S4a). The CPE occurred as early as 24 hours following viral inoculation. In addition, CPE with syncytial cell formation was also observed (Supplementary Fig. S4b). The virus titers are shown in Supplementary Table S5.

## Discussion

Despite vaccination against CDV infection, which has been applied to domestic dogs worldwide, CDV still spreads and continues to cause fatal disease in both wildlife and domestic carnivores. This observation suggested that the virus may have adapted to either escape the anti-viral immunity within the vaccinated hosts or infect to the other susceptible hosts. To date, the genetic diversity of variant CDV strains were better understood by observation of recombinant genetic mutations among the CDV strains. Due to the nature of a single-stranded RNA, high mutation rates caused by the error-prone RNA polymerase frequently occur in the RNA viruses^19^. Although genetic recombination was previously considered to be a rare event for non-segmented negative-sense RNA viruses^26^, it has been suggested that this mechanism is an important contribution to the genetic diversity among the CDV variants^5,20^. In this study, we identified two distinct CDV lineages, the recombinant Asia-1 lineage, and the novel Asia-4 lineage, from the recent Thai CDV isolates. This prompted us to investigate the possible mechanism of the genetic evolution of these viruses.

Our findings indicate that the CDVs circulating in Thailand are diverse. Preliminary analysis by BLASTn demonstrates that the CDV1-3, -5, and -8 TH/2014 are genetically related to the CDV Asia-4 lineage^9^. However, the complete genomic sequence of the previously described CDV Asia-4 was not available. Even the relatively variable region is essential for exploring more distant relationship among the strains, due to a small number of informative sites, evolutionary analysis of partial genes have resulted in low resolution of phylogenetic trees and is limited for exploring the outbreak^27^. Thus, these partial sequences could not be gathered in this study. Recent studies showed that genetic recombination of the CDV viruses had led to its evolution^5,14,20,25^. In this study, we identified one putative and naturally occurring CDV recombinant variant, the CDV4 TH/2014 virus. The parent strains of the CDV4 TH/2014 virus, the CDV Asia-1 strain HLJ1-06 (JX681125) and the CDV America-2 strain 171391–513 (KJ123771) were derived from Chinese fox and American domestic dog, respectively. This recombinant CDV4 TH/2014 virus was speculated to derive from both intra- and inter-genetic recombination events. Interestingly, both putative parent viruses, the CDV HLJ1-06 and CDV 171391–513 viruses, have also been previously described as putative recombinant viruses^5,20^. Therefore, it is possible that those recombinants could later act as putative parents for other recombinant viral progenies. These results imply that the analyses of genetic recombination among the CDV strains should routinely be performed for detection of the novel CDV recombinants. In this study, the recombination breakpoint of the CDV4 TH/2014 virus was identified in the H gene. The result strongly correlated with previous studies and demonstrates that CDV recombination breakpoints have frequently occurred in the H gene^5,13,14^; nevertheless, the recombination events have also been reported in L, P^5,20^ and F genes^28^. Interestingly, the H gene which has been recognized as the most diverse gene^9,29–32^, played an important role in viral attachment to the host cell receptor, SLAM receptor^33,34^. It is not currently known if the recombination might affect the infectivity and pathogenicity of the recombinant virus. Thus, the pathogenicity of the CDV-H gene recombinants should be further explored.

No recombination signals were detected in new Asia-4 lineage which indicates that this lineage is a distinct lineage. The results from evolutionary analysis reveal that the CDVs’ TH/2014 viruses belong to the CDV Asia-4 lineage^9,10^, and to our knowledge, is the first full genome based report on the genetic evolution of the CDV Asia-4 strain.

While the time of the most common ancestor (tMRCA) of CDV based from full genome sequence analysis was estimated at 1855, the analysis based from the H gene sequences yielded the estimated time around 1851. The observed inconsistency of the estimated tMRCA may be a result due to the use of different sequence databases. Previous studies estimated that CDV existed since 1858^25^, while another study suggested that the CDV has tMRCA in 1894^35^. It should be noted that the data sets of those studies were rather small and more geographically and temporally restricted compared to our dataset in which consists of 156 CDV-H gene sequences. The greater number of the analysed sequences and genes certainly affect the interpretation on the existence of CDVs.

The topology of evolutionary analysis of the CDV-H gene revealed that the CDVs’ TH/2014 viruses are grouped into two lineages. Interestingly, most of CDVs’ TH/2014 viruses formed a new clade close to North America (America-2 strains) and existed since 1924 while the other three CDVs’ TH/2014 viruses, including the recombinant one, clustered with the Asia-1 lineage. This finding indicates that the circulating CDVs in Thailand are diverse, unique and existed a long time ago.

RNA viruses are prone to a high frequency of genetic variation caused by various selective pressures^36^. We attempted to assess the nucleotide selection pressure on the individual gene and found that most genes have undergone selective pressure. The results indicate that M gene was the most conserved gene, while the P gene contained the highest negative selection pressure in which is consistent with the previous report^20^. Nonetheless, we were able to isolate CDV from biological specimens and observed the difference in the viral growth curves and titers among the CDVs’ TH/2014 isolates. However, it may be involved with other factors such as viral load, duration of sample collection and tissue culture susceptibility.

In conclusion, we identified the natural intra- and inter-genotypic recombinant CDV strains in which have putative parents of Asia-1 and America-2 viruses. The identification of such genetic recombination events highlights the importance of performing phylogenetic analysis from the whole genome sequence, rather than a single gene region. Although it is clear that full genome analysis is critical for CDV evolutionary analysis, the sequencing of the single H gene serves as a suitable target for CDV’s epidemiological studies. Furthermore, we isolated and characterized a novel CDV Asia-4 lineage. The characterization of genetic diversity among the circulating CDVs is essential for future CDV’s research and disease monitoring. Investigation on the immunogenicity and pathogenicity of the new CDV lineage should be further explored.

## Materials and Methods

### Animal samples

Eight CDV-infected dogs visiting Small Animal Teaching Hospital, Faculty of Veterinary Science, Chulalongkorn University in 2014 were investigated. All dogs showed clinical signs of respiratory, digestive, and nervous systems. CDV infection was initially diagnosed by commercial CDV antigen test kits (Bionote®, Gyeonggi-do, Korea) from nasal and/or conjunctival swabs. After natural moribund, necropsies were performed at Department of Pathology. Fresh tissues including lymph nodes, lung, and brain were collected for molecular diagnosis and viral isolation.

### Reverse transcription polymerase chain reaction (RT-PCR) and whole genome sequencing

Viral RNA was extracted from either conjunctival swabs or tissue homogenates using NucleoSpin Extract Viral RNA Kit (Macherey-Nagel, Düren, Germany). Oligonucleotide primers were specifically designed to regions on whole CDV genome (Supplementary Table S1). The RT-PCR reactions were performed using a one-step RT-PCR system kit (AccessQuick^TM^, Promega, USA). Amplification steps were optimized according to Tm of primers. The PCR products were visualized by 1.5% agarose gel electrophoresis in Tris-borate-EDTA (TBE) and stained with 10% ethidium bromide for observation under a UV illuminator. The amplicons were purified by NucleoSpin Extract II (Macherey-Nagel, Düren, Germany) following to manufacturer’s instructions and submitted for genetic sequencing (1st BASE Pte Ltd, Singapore).

### Genome and phylogenetic analyses

Eight full-length CDV sequences were initially compared to other available CDV sequences deposited in GenBank using nucleotide BLAST (BLASTn) algorithm. The sequences were then aligned with complete CDV sequences gathered from the GenBank using MAFFT alignment version 7 to construct a phylogenetic tree (Supplementary Table S2). A series of transversional models with proportion of invariable sites and substitution model according to Bayesian information criterion in MEGA 7 were tested to obtain the best-fit-model for the phylogenetic tree construction^37^. The best-fit model of full-length CDV genome and its individual genes were described as followed: CDV whole genome = GTR + G + I; P gene = HKY + G; N, M, H and L genes = T92 + G; F gene = GTR + G. The aligned CDV sequences were then used to construct the phylogenetic tree using neighbor-joining and maximum likelihood methods with 1000 bootstrap replicates were performed. The pairwise distances of detected CDV complete genomes were calculated using BioEdit version 7.0.5 (Ibis Biosciences, Carlsbad, USA).

### Recombination analysis

To detect any potential recombination breakpoint of CDVs’ TH/2014 genomes, a cocktail of a statistical method including RDP, GENECONV, BootScan, MaxChi, Chimaera, SiScan and 3Seq with default settings in Recombination Detection Program (RDP) package version 4.0 was used to carry out the recombination events^38^. Because many recombination signals would be detected during the test run and the different algorithms might be inconsistent, any potential breakpoint signals revealed by at least four methods with *p*-values < 0.01 were considered to gain a potentially positive recombination. Initial phylogenetic tree associated with potential recombinant and its putative major and minor parents were defined using RDP 4 package software. The potential recombinant CDV genomes were then subjected to further recombination analysis using similarity plot and bootscan analyses in SimPlot software package version 3.5.1^15^. The putative recombinant served as a query to detect recombination with their parent lineages that derived from the RDP. The recombination analysis was modeled with a window size of 200 bp and step size of 20 bp^16,39^.

### Evolutionary analysis

Fifty-eight full-length CDV and 156 complete CDV-H gene sequences were retrieved from the GenBank database to perform an evolutionary analysis with the potential CDVs’ TH/2014 by using Bayesian Markov Chain Monte Carlo (BMCMC) model implementing on BEAST v.2.4.8^40^. The best-fit substitution model, a general time-reversible substitution model under a strict clock model to account for varied evolutionary rates among lineages and a constant population sizes as priors were implemented by a find-best-fit model for maximum likelihood function on MEGA 7. The coalescent Bayesian skyline tree prior and empirical base frequencies were conducted under three variated models for rate differentiation among branches including a strict molecular clock model (STR), an uncorrelated lognormal relaxed-clock model (LOG) and an uncorrelated exponential relaxed-clock model (EXP), available on BEAST v.2.4.8. Three independent Markov chain Monte Carlo chains were run for 100 million steps and sampled every 10,000^th^ generation, with the first 10% discarded as burn-in. Convergence of parameters was confirmed by calculating the Effective Sample Size (ESS) using TRACER program v. 1.7.0^41^. The log and tree data were combined with the first 10% discarded as burn-in using LogCombiner v. 1.8.3^42^ whenever it presented in another independent run. The results were tracked and displayed by the TRACER. The maximum clade credibility (MCC) trees were annotated using TreeAnnotator v. 1.8.3^43^. The phylogenetic tree with estimated divergence, variable timeline, posterior probability and 95% highest posterior density (HPD) were generated and displayed using FigTree 1.4.2^43^.

### Selection pressure analysis

To determine whether high frequent rate nucleotide substitutions might result in rapid adaptation of RNA viruses, a cocktail of selective pressure tests were assessed on six individual genes of the CDV (N, P, M, F, H and L genes). Non-neutral selection of nucleotide substitutions was calculated by using the ratio value between nonsynonymous (dN) and synonymous (dS) substitutions. The ratio was assessed by phylogenetic reconstruction using Maximum Likelihood model with general reversible nucleotide substitution that is available on the Datamonkey web server (http://www.datamonkey.org). The non-neutral selection was implemented using different models for pervasive individual site measurement^44^ including Single-likelihood ancestor counting (SLAC), Fixed-effects likelihood (FEL) and Fast, Unconstrained Bayesian AppRoximation (FUBAR) on the HyPhy software package^45^. The *p*-value of 0.1 was set at a significant level in all described method. The Bayes factor = 50 was set to estimate the rate of dN and dS within individual codon. The positive selection (adaptive molecular evolution), neutral mutations and negative selection (purifying selection) were defined by using the value of dN/dS > 1, dN/dS = 1, and dN/dS < 1, respectively.

### Virus isolation

Fresh lung tissue samples were homogenized with 1 ml of Dulbecco’s Modified Eagle’s medium (DMEM). After sonication and centrifugation of homogenized samples, the supernatant was collected for virus isolation using the Vero cells expressing canine SLAM (Vero-DST) cell line (Department of Veterinary Microbiology, Faculty of Veterinary Science, Chulalongkorn University). Vero-DST cell line was maintained in DMEM supplemented with 5% fetal calf serum (FCS), penicillin 100 units/ml, streptomycin 100 µg/ml and geneticin (G418) 0.4 mg/ml. Various concentrations of viral suspension were inoculated on 80% confluent Vero-DST in 24-well plate. After a 30-minute incubation, maintenance medium with 10% tryptose phosphate broth (TPB) was added to each well (1 ml/well). CDV-infected cell cultures were maintained at 37 °C in a 4% CO_2_ incubator with daily observation for cytopathic effect (CPE). When CPE was apparent, the viral suspension was collected and kept at −70 °C until used or promptly performed the virus titration in Vero-DST cultured in 96-well plate using a 50% tissue culture infectious dose (TCID_50_) assay and calculated by Reed and Muench method.

### Accession number(s)

Eight full-length CDV sequences have been deposited in NCBI GenBank under accession numbers MH496772 to MH496779 (CDV1-8 TH/2014).

### Ethical Approval

All experimental protocols were approved by the Chulalongkorn University Animal Care and Use Committee (No. 1631002). All procedures were done in accordance to the relevant guidelines and regulations.

## Supplementary information

Supplementary data

## Data Availability

The datasets generated during and/or analysed during the current study are available from the corresponding author upon request.
