# Supplementary material for: Genetic and evolutionary analysis of a new Asia-4 lineage and naturally recombinant canine distemper virus strains from Thailand
Source: Sci Rep. 2019 Mar 1;9:3198. doi: 10.1038/s41598-019-39413-w (PMC6397162; doi:10.1038/s41598-019-39413-w)
Supplement: Supplementary file 1 — Supplementary data [file 41598_2019_39413_MOESM1_ESM.pdf]

Genetic and evolutionary analysis of a new Asia-4 lineage and naturally recombinant canine distemper virus strains from Thailand

Chutchai Piewbang, Araya Radtanakatikanon, Jiratchaya Puenpa, Yong Poovorawan, Somporn Techangamsuwan*

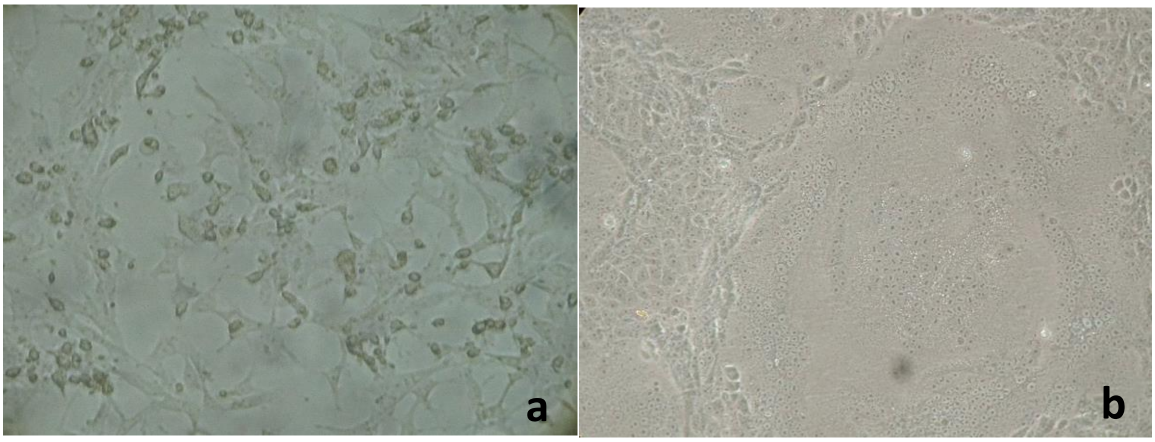

Fig. S4. Cytopathic effect (CPE) after CDV Thai strain inoculation in Vero-DST cell line. Stellate cell formation after 24 hours inoculation Vero-DST with CDV6 TH/2014 (a). and syncytial cell formation (b) after CDV2 TH/2014 inoculation.

Table S1. Sixteen CDV primer sets used for whole genome sequencing

Direction	Primer	Position
**Forward**	5’-ATGGCTAGCCTTCTCAAGAGC-3’	1-21
**Reverse**	5’-CAAATCATTTCAGCAATTCTAGGC-3’	764-741
**Forward**	5’-GAATGAACAAAATATGGCTTGATAT-3’	736-760
**Reverse**	5’-GATTCTCTCTGAGGGCTTTGAG-3’	1867-1846
**Forward**	5’-CTTAGGACCCAGGTCCAACAA-3’	1739-1759)
**Reverse**	5’-GAATCAGTCTCTCCTTTAAGCAG-3’	2843-2821
**Forward**	5’-GCAAAGATGACCCAGAAATGCAA-3’	2635-2657
**Reverse**	5’-CCAGTTGTTCCTTGACACCTG-3’	3765-3745
**Forward**	5’-GGGAGAGATTCAGGAAGAGCA-3’	2995-3015
**Reverse**	5’-GAGCGGGTAACACAGGATTTTC-3’	4283-4262
**Forward**	5’-GGGATTAGTGTTTGCTCTAGGA-3’	4163-4184
**Reverse**	5’-CTCACCTAATTCTGCTTTGGTAC-3’	5495-5473
**Forward**	5’-GGTTAGTCCTATGGTGCATTGG-3’	5287-5308
**Reverse**	5’-CCAGTGGGCAGGTATCGGAG-3’	6537-6518
**Forward**	5’-CAGCCATTTGTAGCCAGAACTC-3’	6289-6310
**Reverse**	5’-AGGTGGATTTATGCACCAATGG-3’	7507-7486
**Forward**	5’-GAAAACTTAGGGCTCAGGTAGT-3’	7051-7072
**Reverse**	5’-AACCGCCCATAAGATGGCAAC-3’	8304-8284
**Forward**	5’-GGTAAAACTTATTTGCTAGTTCCT-3’	7763-7787
**Reverse**	5’-GGGATAAGATAACAAGGTCTGATT-3’	9270-9246
**Forward**	5’-GTCCATCTAGATAGCCCAATTG-3’	9069-9090
**Reverse**	5’-CCATGTCTATCCCGATAACCG-3’	10243-10223

**Forward**	5’-GAAATTCAGGAGATTTTACAGGAC-3’	9960-9983
**Reverse**	5’-GTTTCATTTGCTTTCAAATGATGTC-3’	11497-11473
**Forward**	5’-CCCCTAACTCTCAAATATTCATC -3’	11203-11225
**Reverse**	5’-GTGGTTCCTTAATGCTCTCGC-3’	12458-12438
**Forward**	5’-GGATAGGAAAATTATTATCCCAAG-3’	12179-12202
**Reverse**	5’-TTTCAAAGCCCCAGTTGATTGC-3’	13524-13503
**Forward**	5’-CAGATTGTTGCGTAATACCCATG-3’	13129-13151
**Reverse**	5’-GTAAGGTGAAATCTCTCGTTGTC-3’	14511-14488
**Forward**	5’-GATTAGGAGTCCGGAACTATGAG-3’	14257-14279
**Reverse**	5’-ACCAGACAAAGCTGGGTATGATA-3’	15690-15668

Table S2. Complete CDV sequences retrieving from GenBank used in genetic alignment for phylogenetic analysis.

Accession no.	Strain	Host	Country	Genotype[a]	Year
**AY443350**	00-2601	Raccoon	USA	America-2	2000
**AY649446**	01-2689	Raccoon	USA	America-2	2001
**KJ123771**	171391-513	Dog	USA	America-2	2004
**AF164967**	A75/17	Dog	USA	America-2	N/A
**KJ466106**	CDV SY	Raccoon	China	Asia-1	2012
**KC427278**	Hebei	Mink	China	Asia-1	2008
**HM852904**	MKY-KM08	Monkey	China	Asia-1	2008
**JN896331**	PS	Dog	China	Asia-1	2010
**JX681125**	HLJ1-06	Fox	China	Asia-1	2006
**AB474397**	007LM	Dog	Japan	Asia-2	N/A
**AB476401**	011C	Dog	Japan	Asia-2	N/A
**AB476402**	50Con	Dog	Japan	Asia-2	N/A
**AB475097**	M25CR	Dog	Japan	Asia-2	N/A
**AY445077**	98-2645	Raccoon	USA	America-1	1998
**AY542312**	98-2646	Raccoon	USA	America-1	1998
**AY446011**	98-2654	Raccoon	USA	America-1	1998
**MF041963**	CDV-06	Wolf	Ethiopia	Europe/wildlife	2016
**KP677502**	Louguanthai	Panda	China	Asia-1	2015
**KM280689**	Uy251	Dog	Uruguay	Europe/South America-1	2012
**KY971532**	WT02SA	Hyena	Africa	South Africa	2017

[a] Genotype classification based on complete H gene of the CDV.

N/A: No available data

Table S3. Pairwise nucleotide identity of complete CDV sequences.

**AY466011_CDV_Raccon_isolate_98-2654_America-1**																												
**AY445077.2_CDV_Raccon_isolate_98-2645_America-1**	0.000																											
**AY542312.2_CDV_Raccon_isolate_98-2646_America-1**	0.001	0.001																										
**AF164967.1_CDV_Dog_isolate_A75/17_America-2**	0.029	0.029	0.028																									
**EU716337.1_CDV_Dog_isolate_164071_America-2**	0.029	0.030	0.030	0.004																								
**KJ123771.1_CDV_Dog_isolate_171391-513_America-2**	0.036	0.037	0.036	0.012	0.009																							
**AY649446.1_CDV_Raccon_isolate_01-2689_America-2**	0.028	0.028	0.028	0.013	0.013	0.021																						
**AY443350.1_CDV_Raccon_isolate_00-2601-America_-2**	0.026	0.026	0.026	0.016	0.017	0.024	0.014																					
**MF041963.1_CDV_Wolf_isolate_CDV06_Europe_wildlife**	0.057	0.057	0.057	0.042	0.043	0.049	0.049	0.049																				
**MH496775_CDV_Dog_isolate_CDV4_TH/2014**	0.052	0.052	0.052	0.034	0.036	0.042	0.043	0.043	0.014																			
**KJ466106.1_CDV_Raccon_isolate_SY_Asia-1**	0.055	0.055	0.055	0.040	0.041	0.048	0.047	0.047	0.013	0.009																		
**MH496779_CDV_Dog_isolate_CDV6_TH/2014**	0.055	0.055	0.054	0.038	0.040	0.046	0.045	0.045	0.012	0.011	0.010																	
**KC427278.1_CDV_Mink_isolate_Hebei_Asia-1**	0.054	0.054	0.054	0.039	0.040	0.046	0.045	0.046	0.013	0.012	0.011	0.009																
**JN896331.1_CDV_Dog_isolate_PS_Asia-1**	0.047	0.047	0.047	0.031	0.033	0.039	0.038	0.038	0.012	0.012	0.011	0.009	0.009															
**MH496776_CDV_Dog_isolate_CDV7_TH/2014**	0.054	0.054	0.054	0.038	0.040	0.046	0.045	0.045	0.014	0.014	0.013	0.011	0.011	0.011														
**JX681125.1_CDV_Fox_isolate_HLJ1-06_Asia-1**	0.055	0.055	0.055	0.043	0.044	0.050	0.048	0.049	0.018	0.018	0.017	0.016	0.015	0.012	0.017													
**KP677502.1_CDV_Panda_isolate_Louguantai_1_Asia-1**	0.058	0.058	0.058	0.042	0.044	0.049	0.048	0.049	0.021	0.020	0.020	0.018	0.018	0.012	0.019	0.023												
**HM852904.1_CDV_Monkey_isolate_MKY-KM08_Asia-1**	0.055	0.055	0.055	0.040	0.042	0.048	0.046	0.047	0.029	0.026	0.026	0.025	0.025	0.020	0.026	0.031	0.028											
**MH496772_CDV_Dog_isolate_CDV1_TH/2014**	0.055	0.055	0.055	0.038	0.041	0.046	0.045	0.045	0.057	0.052	0.054	0.054	0.053	0.047	0.054	0.057	0.057	0.056										
**MH496773_CDV_Dog_isolate_CDV2_TH/2014**	0.055	0.055	0.055	0.039	0.041	0.047	0.046	0.046	0.058	0.052	0.055	0.054	0.054	0.048	0.055	0.057	0.058	0.057	0.003									
**MH496777_CDV_Dog_isolate_CDV8_TH/2014**	0.055	0.055	0.055	0.038	0.040	0.046	0.046	0.046	0.057	0.052	0.055	0.054	0.054	0.048	0.054	0.057	0.058	0.056	0.003	0.002								
**MH496778_CDV_Dog_isolate_CDV5_TH/2014**	0.055	0.055	0.055	0.040	0.041	0.047	0.046	0.047	0.058	0.053	0.056	0.054	0.054	0.048	0.055	0.058	0.059	0.057	0.009	0.008	0.009							
**MH496774_CDV_Dog_isolate_CDV3_TH/2014**	0.054	0.054	0.054	0.038	0.040	0.046	0.045	0.045	0.057	0.051	0.054	0.053	0.053	0.047	0.054	0.057	0.058	0.056	0.012	0.011	0.012	0.012						
**KM280689.1_CDV_Dog_isolate_Uy251_Europe/SouthAmerica-1**	0.052	0.052	0.052	0.039	0.040	0.045	0.043	0.043	0.056	0.052	0.055	0.054	0.053	0.047	0.054	0.057	0.056	0.055	0.054	0.055	0.055	0.055	0.055					
**AB474397.1_CDV_Dog_isolate_007Lm_Asia-2**	0.052	0.052	0.052	0.039	0.040	0.046	0.042	0.044	0.059	0.054	0.057	0.056	0.056	0.049	0.056	0.059	0.060	0.057	0.056	0.057	0.056	0.057	0.056	0.056				
**AB476402.1_CDV_Dog_isolate_50Con_Asia-2**	0.053	0.053	0.052	0.040	0.042	0.047	0.046	0.046	0.059	0.054	0.057	0.056	0.055	0.049	0.056	0.058	0.059	0.056	0.055	0.056	0.056	0.056	0.055	0.056	0.009			
**AB476401.1_CDV_Dog_isolate_011C_Asia-2**	0.054	0.054	0.054	0.041	0.043	0.048	0.047	0.047	0.060	0.055	0.058	0.057	0.057	0.050	0.057	0.060	0.060	0.057	0.057	0.057	0.057	0.057	0.056	0.057	0.007	0.003		
**AB475097.1_CDV_Dog_isolate_M25CR_Asia-2**	0.054	0.054	0.054	0.041	0.043	0.048	0.047	0.047	0.060	0.055	0.058	0.057	0.057	0.050	0.057	0.060	0.060	0.058	0.056	0.057	0.057	0.057	0.056	0.057	0.007	0.003	0.000	
**KY971532.1_CDV_Hyena_isolate_WT02SA_South_Africa**	0.057	0.057	0.057	0.044	0.046	0.052	0.050	0.050	0.061	0.058	0.060	0.059	0.059	0.052	0.059	0.062	0.062	0.062	0.061	0.061	0.061	0.061	0.060	0.059	0.056	0.054	0.055	0.055

Table S4. Evidence for positive and negative selection using various detection methods

CDV genome	Selection pressure analysis	SLAC	FEL	FUBAR
H gene	Positive selection	0	4	3
	Negative selection	47	125	134
	Overall dN/dS	0.240	0.228	0.177
N gene	Positive selection	0	0	0
	Negative selection	30	90	143
	Overall dN/dS	0.094	0.087	0.102
F gene	Positive selection	0	8	8
	Negative selection	26	95	100
	Overall dN/dS	0.296	0.280	0.203
P gene	Positive selection	0	2	10
	Negative selection	16	56	42
	Overall dN/dS	0.362	0.336	0.524
M gene	Positive selection	0	0	0
	Negative selection	13	55	73
	Overall dN/dS	0.047	0.039	0.051
L gene	Positive selection	0	1	1
	Negative selection	139	398	278
	Overall dN/dS	0.097	0.086	0.102

Table S5. Viral titer of individual CDV's TH/2014 strain.

CDV strain	Log 10 $TCID_{50}$ /ml
CDV1 TH/2014*	$10^{2.75}$
CDV2 TH/2014*	$10^{3.5}$
CDV3 TH/2014*	10^{5}
CDV4 TH/2014	$10^{2.167}$
CDV5 TH/2014*	$10^{2.875}$
CDV6 TH/2014	10^{3}
CDV7 TH/2014	$10^{2.5}$
CDV8 TH/2014*	$10^{2.625}$

* The strains were classified as CDV Asia-4.
